# Pharmacology of spinal interventions: review of agents used in spine pain procedures

**DOI:** 10.3389/fpain.2024.1408905

**Published:** 2024-10-09

**Authors:** Ericson John V. Torralba, Robert F. Short, Jeffrey B. Travers, John M. Mathis

**Affiliations:** ^1^ Department of Interventional Radiology, UCLA Medical Center, UCLA David Geffen School of Medicine, Los Angeles, CA, United States; ^2^ Boonshoft School of Medicine, Wright State Univeristy, Dayton, OH, United States; ^3^ Department of Pharmacology and Toxicology, Boonshoft School of Medicine, Wright State University, Dayton, OH, United States; ^4^ Department of Therapeutic and Diagnostic Imaging, Dayton VA Medical Center, Dayton, OH, United States

**Keywords:** spine interventional procedures, pharmaceuticals, spine, radiology, spine procedures

## Abstract

Spine procedures are commonly performed to diagnose and treat various spinal conditions, ranging from degenerative disc disease to vertebral fractures. These procedures often involve the use of pharmaceutical agents to enhance the efficacy of the intervention and improve patient outcomes. This review provides an overview of the pharmaceuticals commonly utilized in spine procedures, including corticosteroids, anesthetics, antibiotics, radiographic contrast, neurolytic agents, and materials used in kyphoplasty and vertebroplasty. This review summarizes the utilization of these pharmaceutical agents in spine procedures in an effort to optimize patient outcomes. Understanding the pharmacological properties and appropriate uses of these pharmaceuticals is essential for interventionalist and healthcare providers involved in the care of patients undergoing spinal interventions.

## Introduction

Pharmaceutical innovation in medicine has substantially advanced the delivery of spinal therapy and propagated the development of agents to support the latest developments in interventional spinal procedures. The progressive historical use of corticosteroids, anesthetics, antibiotics, analgesics, adjuvant analgesics, radiographic contrast, and materials used for bone augmentation (vertebroplasty/kyphoplasty) in interventional radiology has also paved the involvement of other specialties such as anesthesia, physical medicine and rehabilitation, pain management, orthopedics, and neurosurgery. In this review, we explore the historical relevance, proposed mechanisms of actions, indications, complications, and various details regarding common pharmaceuticals utilized in interventional spinal procedures.

## Corticosteroids

Since the 1960s, corticosteroids have been utilized for the treatment of pain associated with spine disease (1, 2). However, corticosteroid use became controversial due to the rising number of complications concurrent with epidural and intrathecal injections. Adverse reactions reported between 1956 and 1991 involved arachnoiditis, chemical meningitis, and subarachnoid hemorrhage. Despite the numerous negative drug experience reports to the Food and Drug Administration, there was no evident decline in its usage (3). It was later revealed that adverse side effects were primarily the result of injection of corticosteroids into the intrathecal space which has been rarely used since. These medications are now recognized to have specific side effects when injected for spinal procedures and require prompt awareness by the clinician so that they may highlight potential consequences to the patients they treat (4, 5).

**Figure 1:**
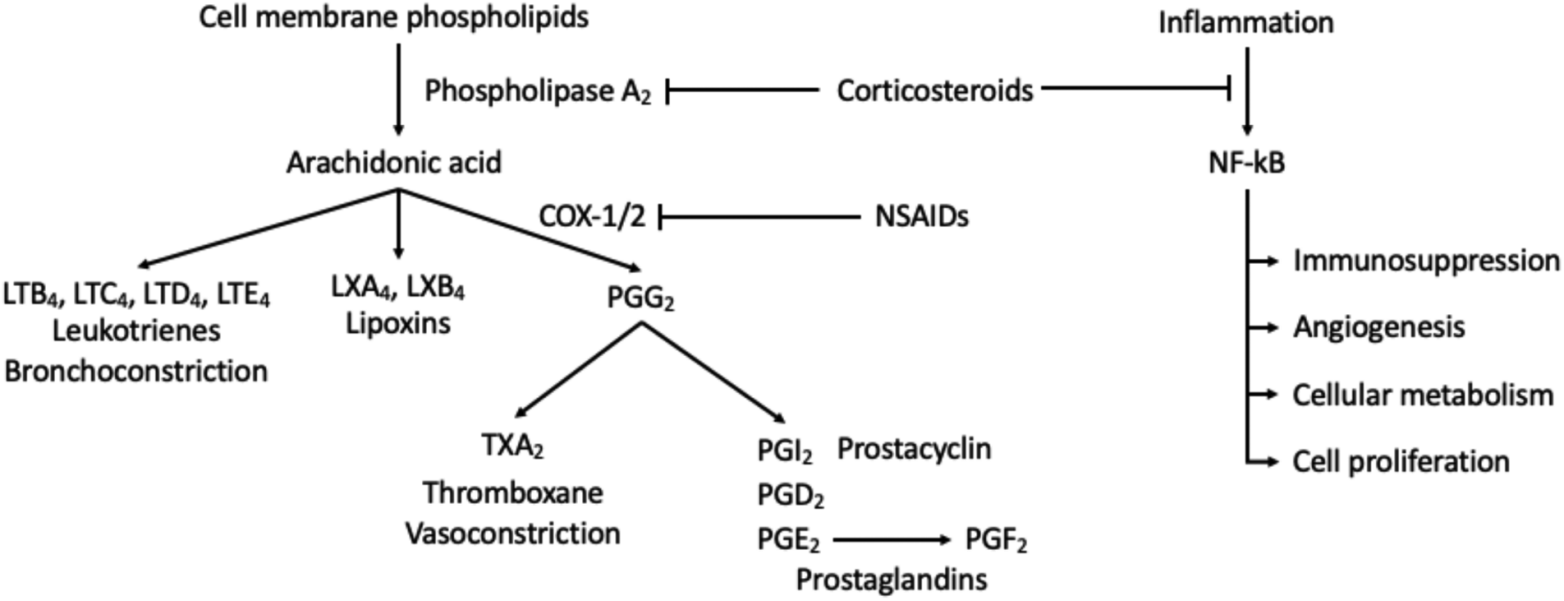
Activation of inflammatory and immune mediators. Corticosteroids inhibit activation of phospholipase A_2_ which decreases the production of downstream response. This mechanism thereby inhibits the immunosuppression, angiogenesis, cellular metabolism, cell proliferation, and anti-apoptotic effects of NF-kB.

Disc, tissue, or nerve injuries associated with the spine are believed to undergo the inflammatory and pain response mediated by phospholipase A2, resulting in the production of arachidonic acid. Corticosteroids relieve pain and inflammation through the inhibition the phospholipase 2 cascade, reducing the formation of the arachidonic acid and downstream inflammatory and immune mediators (6). Corticosteroids have similar structure to and mimic the endogenous effects of cortisol, thus, further alters the levels of prostaglandins, thromboxane, leukotrienes, and proinflammatory cytokine (Figure 1). Overall, this cascade results in immunosuppressive, vasoconstrictive, antiproliferative and anti-inflammatory effects on the body. In addition, corticosteroids are proposed to alter nerve transmission in nociceptive C fibers, decreasing vasal permeability leading to pain reduction (7, 8). On a molecular level, glucocorticoids have been shown to interfere with cytosolic signaling molecules such as AP-1, STAT5, NF-kB, CREB, and others to induce proapoptotic states of T lymphocytes (9). Before the discovery of glucocorticoid molecular pathways, glucocorticoids have been and will be consistently utilized for management of pain in regards to the spine therapy (Table 1).

**Table 1:** Recommended dosages and types of steroids.

Steroid	Dose (mg)	Type
Methylprednisolone Acetate (Depo-Medrol)	40 mg/ml 80 mg/ml	Particulate
Triamcinolone (Kenalog)	40 mg/ml	Particulate
Betamethasone sodium phosphate and betamethasone acetate (Celestone)	6 mg/l (3 mg/ml +3 mg/ml)	Mixed particulate and non-particulate
Dexamethasone sodium phosphate (Decadron)	6 mg/ml	Non-particulate

Recommended Dosages for epidural steroid injections (particulate/non-particulate).

There are two types of injectable corticosteroids, particulate and non-particulate. Particulate corticosteroids are known to have slow, long-term anti-inflammatory effects whereas, non-particulate corticosteroids tend to have a rapid onset with brief effects (10). Particulate and non-particulate qualities of corticosteroids are given in Table 2.

**Table 2:** Qualities of particulate and nonparticulate steroid preparations.

Particulate	Non-particulate
Aggregation of particles	Little to no aggregation of particles
Increased embolus formation	Water soluble
Increased stroke potential	Short duration of action

Qualities of particulate and non-particulate steroid preparations.

Methylprednisolone acetate (Depo-Medrol, Pharmacia-Upjohn, Chesterfield, MO, USA) is a particulate corticosteroid commonly used for spine injections and is available in 40- and 80-mg/ml doses prepared with polyethylene glycol. When compared to hydrocortisone, methylprednisolone has a relative potency of approximately 4–5 times greater. Derby et al. found methylprednisolone particles to be the largest of particulate corticosteroids and densely packed but smaller than the size of red blood cells regardless of mixture with local anesthetic and contrast medium. Its particulate nature suggests the potential to form an embolus and occlude small arterioles when injected intra-arterially leading to ischemia or infarction of neural tissue (10, 11).

A similar corticosteroid, triamcinolone (Kenalog, Bristol-Myers Squibb, New York, NY, USA), is also particulate in nature and available in 40-mg/ml and tends to form aggregates mirroring that of methylprednisolone. Dosage for triamcinolone is similar to methylprednisolone with no difference in efficacy between the two (12).

An alternative injectable corticosteroid is a combination mixture of betamethasone sodium phosphate and betamethasone acetate (Celestone Soluspan, Schering, Kentworth, NJ, USA). It is available in a 6 mg/ml dose containing 3 mg/ml of betamethasone sodium phosphate and betamethasone acetate. Approximately 20 times the strength of hydrocortisone, its particulate and the non-particulate combination makes it less susceptible to aggregation and arachnoiditis. Betamethasone sodium phosphate's soluble characteristics allow for a rapid onset while its acetate counterpart provides a depot effect (13). Darby et al. report betamethasone particles to be rod-shaped and the smallest of the particulate corticosteroids; however, extensive aggregations were observed to be 12 times greater than the size of red blood cells (10).

The particulate qualities in these corticosteroids increase stroke potential, posing a concern for their use in transforaminal procedures where the intra-arterial injection is possible. Albeit rare, the catastrophic neurologic complications associated with particulate corticosteroids warrant the advocacy of some clinicians to only use nonparticulate corticosteroids in transforaminal procedures (11).

Dexamethasone sodium phosphate is the nonparticulate substitute for injectable corticosteroids in epidural procedures and is small enough that it does not carry the risk of embolic infarction secondary to aggregation. Thus, it has gained increasing popularity among clinicians despite its potential to be washed out of their target region (12). Moreover, recent literature reviews have observed dexamethasone to have equal or close to equal efficacy to that of particulate steroids without the added risk of neurological complications.

In 2014, the Food and Drug Administration published a warning and safety announcement that required manufacturers to include the neurological side effects of injected corticosteroids onto their package insert. They stated that the effectiveness and safety of the drugs for this [epidural spinal injection] have not been established, and the FDA has not approved corticosteroids for such use. This announcement included the CDC's previous concern for a multistate outbreak of fungal infection after the use of methylprednisolone acetate (MPA) from a single compounding pharmacy. In September of 2012, a patient was diagnosed with culture-confirmed *Aspergillus fumigatus* meningitis 46 days after an epidural steroid injection. In less than 10 days, an additional 8 patients were clinically diagnosed with meningitis. The FDA had identified 137 cases and 12 deaths associated with the outbreak with almost 14,000 persons potentially exposed to this contamination. The investigation yielded four categories for potential significant unwanted side effects: (1) fungal meningitis (2) basial stroke (3) spinal meningitis and (4) septic arthritis in the 137 persons. At the time, the manufactured MPA was recalled by the NECC and treatment for the infected persons were initiated (14). Since the release of the warning and safety announcement in 2014, several professional societies responded with concern that the FDA had not provided insight on the differences between transforaminal procedures vs. interlaminar approaches, and particulate vs. non-particulate forms of corticosteroids and the associated unwanted side effects, therefore providing negative insight regarding its use (11). Despite this announcement, the utilization of corticosteroids for epidural spinal injections has been a continued and prevalent practice for decades (11, 15).

## Anesthetic agents

Since the discovery of cocaine in the 1860s as a natural, highly addictive local anesthetic, the progressive development and use of its various synthetic analogs have provided patients with cutaneous analgesia during spinal and pain management injection procedures. Procaine, the first synthetic local anesthetic, was developed in 1904 and found to have a short duration of action, less potency, and delayed onset of action when compared to cocaine. Lidocaine was developed in 1943 and bupivacaine in 1957. Ropivacaine was developed in 1996 and serves to be more potent than lidocaine with less cardio- and chondrotoxicity (15, 16).

Trauma to skin, muscle, joints, bones, and viscera results in a local inflammatory response that activates nociceptors, free nerve endings of A*δ* fibers, causing depolarization of voltage-gated sodium channels, which in turn produce the sensation of pain. Local anesthetics function by inhibiting these voltage-gated sodium channels found on the neuronal cell membranes. By blocking the inflow of sodium into these cells, an action potential cannot be generated and thus results in the halting of the electrical impulse conduction. In addition, the small A*δ* fibers experience calcium channel blockade with the smallest amount of anesthetic resulting from the blocking of 3 sodium channel receptors. This mechanism contributes to the theory that minute nociceptive receptors are targeted readily due to their size compared to the larger sensory or motor fibers, where more than 3 consecutive sodium or calcium channels are not targeted (2).

**Figure 2:**
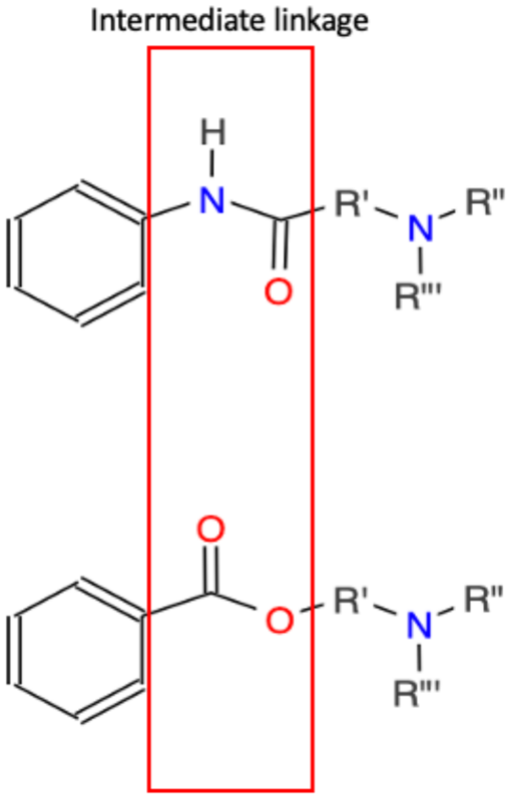
Basic structure of local anesthetics. Top intermediate linkage is an amide while the bottom intermediate linkage is an ester. These linkages join an aromatic ring and tertiary amine. Variations and extended functional groups create various local anesthetics.

The structure of local anesthetic drugs is composed of a lipophilic aromatic group, an intermediary link (an ester or amide), and a hydrophilic amine group (Figure 2). By increasing the length of carbon chains that are attached to either the aromatic ring, intermediary link, or amine group, one can increase the potency, action of duration, and lipid solubility (17). However, changes in their structure also contribute to their metabolism and allergic potential. Esters may be hydrolyzed more rapidly by pseudocholinesterase leading to the formation of para-aminobenzoic acid (PABA), a metabolite associated with anesthetic allergic reactions. On the other hand, anesthetics with amide groups do not form PABA metabolites and instead undergo hydroxylation, amide hydrolysis, and N-dealkylation. Amide-based anesthetics are metabolized slower and may accumulate in the presence of hepatic disease solubility (17).

Amides are the most utilized local anesthetics in spine injection procedures, especially lidocaine and bupivacaine. Lidocaine has a quick onset, short duration of action, and lower potency when compared to bupivacaine. However, the cardiac toxicity related to bupivacaine is notably higher, thus ropivacaine was developed with similar potency and less cardiotoxic side effects (18). Typical doses for bupivacaine are available in 0.5–2.0 ml in concentrations of 0.25%, 0.5% and.75% and Ropivacaine is available in concentrations of 0.2% and 0.5%. The common local anesthetics are given in Table 3 and include their structural classification, the onset of duration, dosage, elimination half-life, and duration of action (Table 3). Despite the concern for potential cardiotoxic side effects, percutaneous spine interventions require low doses, never warranting the need to approach maximum doses associated with such adverse reactions (2).

**Table 3:** Physiochemical characteristics of various local anesthetics.

	Structural Classification	Onset	Dosage mg/kg (with vasoconstrictor)	Dosage mg/kg (without vasoconstrictor	Elimination half-life (min)	Duration of action (h)
Cocaine	Ester	Fast	1.5 (topical)	-	100	0.5–1
Chloroprocaine	Ester	Fast	11	14	6	0.5–1
Prilocaine	Ester	Fast	6	8	100	0.5–1
Tetracaine	Ester	Slow	1.5	3	2–4	1.5–10 w/epi
Lidocaine	Amide	Fast	3	7	100	0.57–1.5 w/epi
Mepivacaine	Amide	Fast	5	7	115	1–2 w/epi
Bupivacaine	Amide	Slow/moderate	2	2	210	1–8
Ropivacaine	Amide	Moderate	3	3	120	0.5–8
Levobupivacaine	Amide	Slow/moderate	2	2	210	9–12

The common local anesthetics and their structural classification, the onset of duration, dosage, elimination half-life, and duration of action.

Interestingly, Manchikanti et al’s one-year follow-up of a randomized, double-blind controlled trial for lumbar facet nerve blocks demonstrated bupivacaine injections providing pain relief for a median duration of 15 weeks despite the anesthetic’s effect of 6–7 h (19, 20). This mechanism is unknown and warrants further studies.

Local anesthetics are commonly used in spinal injections, allowing the clinician to provide short-term pain relief. A common complaint amongst patients is the burning sensation upon local anesthetic injection. This is primarily due to its acidic nature and may be buffered by the addition of sodium bicarbonate in a 10:1 lidocaine: sodium bicarbonate mixture (21).

## Antibiotics

Antibiotic use during image-guided interventions considers the possible inoculation of bacteria into the bloodstream. Therefore, antibiotic prophylaxis for image-guided interventions aims to clear bacterial contamination from needles, catheters, or wires into the bloodstream, preventing sepsis or abscess formation (22).

Antibiotics are only required for a few procedures in spine image-guided procedures. Discography, intradiscal electrothermal treatment, percutaneous discectomy, vertebroplasty and kyphoplasty, implanted pumps, and stimulators are examples of these procedures (2). Thus, common epidural steroid injection procedures do not usually require the use of antibiotics. However, there have been multiple case reports that utilize computed tomography guidance for a biopsy in patients with suspected malignancy or infections of the spine and epidural abscesses that require the use of antibiotics (23–28). Despite the minimally invasive nature of these types of procedures, it is common practice to provide antibiotic coverage to decrease the probability of seeding bacteria into areas around foreign bodies (pumps and implants) or poorly vascularized sites.

Prophylactic antibiotics are the administration of antibiotic agents before the incision or skin puncture. According to The Joint Commission recommendations, intravenous (IV) antibiotics are to be administered within 1 h of an incision and a repeat dose if 2 h have elapsed since the initial dose.

In cases where prophylactic antibiotics are required, a broad-spectrum antibiotic with little to no penicillin cross-reactivity is often suitable. Cefazolin, a first-generation cephalosporin, is an adequate selection due to its minimal penicillin cross-reactivity. Generally, 1–2 g dosages are given IV 1 h before the procedure. If penicillin-allergy is of concern, Vancomycin can also be utilized according to pharmacy protocol (22). Penicillin allergy is not uncommon and has been reported to be found in as many as 22% of the general population (29). Despite patient reports of allergic symptoms such as a rash, hives, abdominal pain, or nausea, these may not be true hypersensitivities (30). Symptoms of concern include bronchospasms, pulmonary edema, laryngospasm, and hypotension which are uncommon (31). These general principles are suitable for percutaneous image-guided procedures such as spinal biopsies, vertebroplasty, kyphoplasty, or discography. A mixture of antibiotics with contrast (for discography) or cement (vertebroplasty or kyphoplasty) has been employed but is not advantageous over IV antibiotic agents alone (32).

Fluoroquinolones like ciprofloxacin, levofloxacin, and moxifloxacin are other commonly used antibiotic agents in image-guided procedures. When there is a concern for allergy or the lack of IV access, this antibiotic class may be a possible alternative. However, keep in mind the increased risk of tendinopathy or tendon rupture that is associated with fluoroquinolones which has gathered enough concern to warrant a black box warning by the US Food and Drug Administration (33).

Of note, there has been a rapid emergence of drug-resistant bacteria that has outpaced the development of new antibiotic agents warranting the World Health Organization to release a warning to clinicians about antibiotic resistance (34). Appropriate use of antibiotic agents with the narrowest spectrum of activity can provide sufficient protection and can limit the progression of antibiotic resistance (22).

## Analgesics

The use of analgesics for image-guided spine procedures is reserved for intraoperative sedation and postprocedural pain flairs. Analgesia is the relief of pain without the intentional production of an altered mental state (35). Although, the utilization of moderate sedation may have an analgesic effect. This is often utilized in spinal interventional procedures of percutaneous vertebroplasty or kyphoplasty (2). Moderate sedation for these patients is described as a drug-induced depression of consciousness whereby patients can purposefully respond to verbal commands without significant effect on cardiopulmonary function (36). Anesthesiologist-driven sedation may also be utilized in patients who are refractory to the above methods. Drugs that are used for pain management during the peri- and postprocedural period include opioids, nonsteroidal anti-inflammatory drugs (NSAID), or a combination of both (2).

Opioids are considered the most potent form of analgesics used for postprocedural pain with various routes of administration and are often reserved for severe pain. Common drugs found in this class are Morphine, Fentanyl, Hydromorphone (Dilaudid), methadone, and meperidine (Demerol). These opioids are seldom prescribed by the spine interventionist; however, they may be used on a case-by-case basis, tailored for each patient, with proper documentation and understanding of these drugs.

If pain is mild to intermediate, non-opioid or NSAID use for postoperative pain management may be used in combination with a weak opioid (codeine, hydrocodone, dihydrocodeine, or oxycodone) or alone. It is recommended that these drugs are prescribed in a stepwise fashion beginning with NSAIDs, progressing to weak opiates before the stronger opioids. NSAIDs function by inhibition of COX-1 or COX-2, found in the inflammatory cascade (Figure 1). Cautioned use of NSAIDs applies when there is a concern for the developing gastropathy or gastrointestinal bleeding. Ketorolac is an NSAID available for IV administration and very effective for short-term use for pain management during or after procedures. Its use should not exceed several days and if the continuous use of NSAIDs is warranted, an oral alternative is suggested (37). Ketorolac dosing for patients under the age of 65 prompts 30 mg every 6 h with a maximal dosage of 120 mg. For patients above the age of 65, renal dysfunction, or weighing less than 50 kg, 15 mg every 6 h with a maximal dosage of 60 mg is sufficient for pain relief (2).

A combination of NSAIDs and weak opioids can be utilized for the management of intermediate pain. Codeine, hydrocodone, dihydrocodeine, or oxycodone are available in combination with aspirin, acetaminophen, or ibuprofen. Hydrocodone preparations, like Vicodin or Lortab, are commonly used for postprocedural moderate pain management with intermediate potency between codeine and oxycodone. Oxycodone is available as a combination drug, Percocet (acetaminophen) or Percodan (aspirin), or by itself, Roxicodone, and is very effective.

Patient's undergoing spinal intervention commonly present with baseline pain and undergo these procedures (epidural spinal injections) to alleviate or decrease pain levels. The interventionalist aims to mitigate the use of these analgesics in the long term (37).

## Adjuvant analgesics

Pain is often readily alleviated using opioids, NSAIDs, or a combination of both (2). However, if these methods of pain management are not sufficient due to the presence of neuropathic pain, antidepressants or anticonvulsants may be employed. These drugs are called adjuvant analgesics, which were originally developed for a primary indication other than pain (38).

When the pain is neuropathic, which is described as constant and burning, the use of antidepressants provides relief. The mechanism of antidepressants is proposed to block the reuptake of serotonin, noradrenaline, and dopamine into the presynaptic nerve terminal; however, they may also have cholinergic, α-adrenergic, and histaminic blockade (38). These analgesic effects are independent of their antidepressant properties and aid to relieve peripheral neuropathic pain. Among the antidepressants, the serotonin-norepinephrine reuptake inhibitor (SNRI), duloxetine, has the strongest evidence for analgesic efficacy (39). Another group of antidepressants are tricyclic antidepressants (TCAs) which have shown to be useful for dysesthetic pain (40). However, these drugs are characterized by a list of unwanted side effects such as anticholinergic symptoms, orthostatic hypotension, sedation, and impaired cardiac conduction.

Other first-line therapies for neuropathic pain include the use of gabapentinoid drugs or topical therapies. Gabapentinoid drugs such as gabapentin and pregabalin inhibit nociceptive neurons by binding to the N-type voltage-gated calcium channels and serve as significant agents under adjuvant analgesics for the treatment of chronic neuropathic pain. Topical therapies are also available for the treatment of focal neuropathic pain. These include topical NSAIDs, local anesthetics, and Capsaicin. Capsaicin is naturally occurring and able to inhibit primary sensory neurons in the periphery. It comes in low and high-dose creams or patches and has been utilized for postherpetic neuralgia or peripheral neuropathic pain (39).

Adjuvant analgesics may be useful in opioid-refractory pain syndromes and can be combined with topical options, especially when the pain is focal or regional. Although evidence is limited, other adjuvant analgesics such as cannabinoids, benzodiazepines, or α-adrenergic agonist have shown promise as alternative options and requires further studies to judge their efficacy.

## Radiographic contrast

The two primary types of contrast media utilized in interventional procedures are differentiated by being ionic or non-ionic. The clinician has a vast array of choices for contrast media during their minimally invasive spinal procedure however, there is a concern for the allergic potential of certain types of contrast agents. Allergy to contrast agents primarily undergo acute hypersensitivity reactions and vary in severity. Severe reactions include convulsions, pulmonary edema, hypotensive shock, and cardiopulmonary arrest (41). Therefore, premedication is indicated for those with a known allergy to contrast media agents. According to the American College of Radiology, pretreatment can be prednisone-based or methylprednisolone-based. Prednisone-based elective premedication consists of 50 mg of oral prednisone at 13, 7, and 1 h before contrast administration plus 50 mg of diphenhydramine IV, intramuscular, or orally 1 h before contrast administration. The methylprednisolone-based premedication utilizes 32 mg of oral methylprednisolone 12 and 2 h before contrast medium administration. 50 mg of diphenhydramine may also be added optionally. These two have never been formally compared in a study, however, are both historically efficacious. Pediatric doses vary by weight.

Allergic reactions toward nonionic contrast are approximately 3% or less and ionic contrast media is assumed to be higher. Routine use of nonionic contrast like Isovue, Omnipaque, Optivist, and Optiray is found to be effective and safe for facet and sacroiliac joint injections (2). If there is a probability of injection into the thecal sac, proper use of an approved contrast medium is highly recommended. Clear identification found on respective package inserts can point to whether the contrast agent is suitable for intrathecal use.

## Neurolytic (cytotoxic) agents

Cytotoxic agents in the realm of image-guided spinal procedures are primarily utilized neurolysis in patients with visceral pain, leading to the destruction of nerve cells. This can be achieved by injection of chemicals, like phenol or alcohol, or thermal destruction, by radiofrequency or cryoablation. Chemical neurolysis ultimately leads to the disruption of sympathetic ganglia through obliterative fibrosis and although is a common procedure, thermal neurolysis serves as a primary choice of modality for image-guided spinal procedures due to risks associated with nontarget destruction due to unintentional migration of liquid agents (2, 42, 43). The use of neurolytic agents for selective, iatrogenic destruction of neural tissue for pain is commonly paired with image guidance for increased precision in spinal interventional pain management. Moreover, it is common practice to first perform a targeted nerve block using a liquid anesthetic of similar volume at the intended site of chemical neurolysis.

Nonselective neurolytic chemical agents like phenol, ethyl alcohol, hypertonic saline, ammonium salts, and chlorocresol have been previously used for pain relief in spinal image-guided interventions (43, 44). The more commonly used phenol and alcohol cause protein denaturation within cells through Wallerian degeneration which lasts from 3 to 6 months and provides lasting pain relief. Alcohol is available at 50%–95% concentrations but causes severe pain to patients at higher concentrations, therefore concentrations above 50% are not commonly used without sedation. Concentrations below 50% have been shown to spare motor nerve targeting when applied peripherally (45). Phenol is available in many concentrations; however, concentrations between 5% and 10% are more commonly used. Of note, phenol is not readily available for injection and must be prepared by the hospital pharmacist (2, 45). Glycerol is available at 50% concentrations along with ammonium, hypertonic solutions, and chlorocresol, though it is not often clinically used.

Complications related to the injection of neurolytic agents are thought to be a result of the uncontrolled spread of the alcohol or phenol into sensitive spaces. Alcohol specifically, is painful and toxic to the vasculature and connective tissue leading to vasospasm and necrosis, respectively. A fearful effect of alcohol is paralysis, with an unpredictable duration, and neuritis. Conversely, phenol is not found to be painful upon injection and is less likely to cause neuritis. However, it has been shown to have the same effect on the vasculature as alcohol (45).

## Materials used for percutaneous vertebroplasty or kyphoplasty

The selection and development of materials for vertebroplasty or kyphoplasty are based primarily on the ability of the cement to mimic bone. Thus, it is essential to consider the mechanical properties of bone and how implanted materials compare. In addition, implant injectability is of concern due to a phenomenon termed liquid-phase migration whereby high-pressure during injections causes poor stability of the injectable formula leading to an excess of water in the cement injection. To solve this problem, their cement design may increase the viscosity of the liquid cement mixture, decrease the permeability of particles, or use a pressure-independent large syringe bores to facilitate easy injectability (46). The interventionalist may also be concerned with the ability of the material to stay at the site of injection, preventing leakage. Therefore, the manipulability of the cement during the procedure in combination with the setting time is of great use to the interventionalist (46). Biocompatibility of the product must not provoke inflammatory responses in the patients whom they are injected into, and thus, is a high consideration when developing these materials. Ideally, the material injected would have the capability to induce bone regeneration. Although only a few substances have been shown to elicit osteoconductive reactions *in vivo*, a material designed to cause bone resorption and formation will be of higher consideration if it can integrate with the host bone and provide the biomechanical aptitude for loadbearing and remodeling (47).

Currently, the various types of materials used for kyphoplasty procedures are acrylic bone cements, calcium sulfate and calcium phosphate bone cement, and composite bone cements (46, 48). The first use of polymethylmethacrylate (PMMA), a classically used acrylic bone cement, on the spine was in 1984 by Deramond and Galibert. Since then, PMMA has been the material of choice for percutaneous vertebroplasty and kyphoplasty (49, 50). PMMAs first commercial application was through the development of Plexiglas during World War II. Since then, PMMA is the most used filling material for vertebroplasty and kyphoplasty primarily because of its low viscosity, ability to perfuse during injection, strength after setting, and price point (46). However, these advantages also serve as a disadvantage in the procedural setting. Its low viscosity allows for easy injectability but can also lead to a leakage complication resulting in compression of the spinal cord, entry of PMMA into the vasculature, and possible pulmonary embolism. PMMA is mixed with a non-ionic liquid contrast and, when injected, has an exothermic polymerization reaction with temperatures up to 110°C during hardening (48). This reaction may cause thermal burns to the surrounding tissue, requiring delayed injection times if the mixture temperature is elevated. PMMA has also been reported to cause extensive bone stiffening and fractures to the adjacent vertebral bones, affecting its efficacy (51). The lack of bone conductivity and bioactivity cause a fibrous tissue layer to develop between PMMA and bone. This inflammatory response is like that of foreign object infiltration and host rejection (47, 48). Overall, PMMA is a highly sought-after material for vertebroplasty and kyphoplasty due to its ability to cure quickly and exhibit high mechanical and compressive strength. Numerous commercially available acrylic bone cements vary in percent PMMA, working time, setting time, viscosity, and strength (Table 4-types of PMMA and variables).

**Table 4:** Equivalent doses of steroids (mg).

Steroid	Dose (mg)
Cortisone	25 mg
Hydrocortisone	20 mg
Prednisone	5 mg
Prednisolone	5 mg
Methylprednisolone	4 mg
Triamcinolone	4 mg
Paramethasone	2 mg
Betamethasone	0.75 mg
Dexamethasone	0.75 mg

Equivalent doses of various steroids.

Another category of bone cements is calcium bone cements (CPCs), which were developed in the early 1980s by Brown and Chow (52). The use of CPCs has gained significant interest due to their ability to integrate with the host bone and facilitate bone remodeling. The mixture of the cement creates a network of calcium and phosphate crystals that resemble bone making it an excellent biocompatible, osteoconductive, and bio resorptive material for vertebroplasty and kyphoplasty (53). In addition, its similarities to physiologic bone remodeling allow it to form a porous structure, further allowing fluid exchange and the ability to participate in biochemical processes for the development of new bone and vascular infiltration (48, 53) Compared to PMMA, CPCs have the advantage of bone conductivity and the lack of heat generation during crystal formation, preventing potential thermal burn injury. In addition, its viscosity allows excellent dispersion capabilities with arbitrary shaping, decreasing the risk for upper and lower vertebral fractures upon injection (48, 52–54) Although CPCs have been found to gradually increase in strength with the formation of woven bone after several weeks, its long setting time and poor injectability is of major concern. This is believed to be due to the separation of solid and liquid phases during cement delivery (46, 54). Further studies regarding the use of viscous solutions or additives to CPC, like strontium, are currently underway to tackle concerns of cohesion and injectability (53). In addition, injection delivery systems are also currently being studied to allow compatibility with difficult-to-inject materials.

A commonly used composite bone cement is Cortoss^™^ and considered to be a low viscosity cement that utilizes a non-volatile liquid monomer with the consistency of toothpaste. This multi-material cement reaction is primarily exothermic, not to the degree of PMMA, and has similar qualities to CPCs such as; good bioactivity, strength over time, high osteoconductive properties, and modulus similar to cancellous bone (46, 55).

Orthocomp, another composite bone cement, is comparable to PMMA but has desirable advantages like Cortoss^™^. It is primarily composed of a glass-ceramic matrix with bioactive and bioresorbable qualities. Of note, it was found to have double the strength and stiffness when compared to PMMA (56).

Ideally, the advancement in the development of bone-like materials can enhance anti-pressure capacity, maintain morphological characteristics like true bone tissue when fractured, and restore the biomechanical attributes of a fractured bone. However, desirable properties of various bone cements belong to the interventionalist. An ideal bone cement will have low curing temperatures, easy injectability, low setting time, excellent osteoconductive properties, excellent biocompatibility, excellent bioactivity, low cost, porous scaffolding, appropriate working time, and easy preparation and handling. Although the list is long, these fundamental properties are necessary to provide a level of safety and efficacy to that of normal bone tissue.

## The role of antiplatelets and anti-coagulation on spinal procedures

Although antiplatelets and Anti-coagulation agents are not primarily used during image-guided spinal procedures, it is not unlikely that the interventionalist will encounter patients taking these medications. Although the risk for bleeding in anticoagulated patients is higher for lumbar punctures or epidural anesthesia, spinal procedures such as myelography, vertebroplasty, or epidural injections carry similar risks for the bleeding or the development of epidural or subdural hematomas (57–60). The American Society of Regional Anesthesia and Pain Medicine has classified pain procedures into three categories: High-Risk, Intermediate-Risk, and Low-Risk procedures, in which spinal procedures are ranked by potential risk for serious bleeding (Table 5) (61). Thus, to minimize bleeding complications, it is imperative that the interventionalist understand the roles of antiplatelet and anti-coagulation on spinal procedures.

**Table 5:** Pain procedure classification by the regional anesthesia and pain medicine guidelines.

High-risk	Spinal cord stimulation trial and implant
	Dorsal root ganglion stimulation
	Intrathecal catheter and pump implant
	Vertebral augmentation (vertebroplasty and kyphoplasty)
Intermediate-risk^a^	Interlaminar epidural steroid injections (all spinal levels)
	Transforaminal epidural steroid injections (all spinal levels)
	Cervical facet medial branch nerve block and radiofrequency ablation
	Intradiscal procedures (cervical, thoracic, and lumbar)
	Sympathetic blocks (stellate, T, splanchnic, celiac, lumbar, hypogastric)
Low-risk^a^	Peripheral nerve blocks
	Sacroiliac joint injections and sacral lateral branch blocks
	Thoracic and lumbar facet medial nerve block and radiofrequency ablation

^a^ Patients with high risk of bleeding due to history of bleeding, multiple anticoagulation/antiplatelet use, liver cirrhosis, advanced liver disease, and advanced renal diseases undergoing intermediate-risk or low-risk procedures should be treated as intermediate or high risk, respectively.

### NSAIDS

As previously discussed, NSAIDS act upon the inflammatory cascade through inhibition of prostaglandin production and ultimately preventing the formation of prostaglandin H_2_ and thromboxane A_2_, leading to decreased platelet aggregation (Figure 1). Aspirin (ASA), an irreversible COX-1 inhibitor, has been noted to be a significant risk factor for the development of epidural hematomas in spinal procedures (58–60). Current literature has shown low-dose ASA to increase the rate of bleeding complications by 1.5× the baseline rate (62). Thus, current recommendations by the ASRA Pain Medicine are to discontinue ASA for a minimum of 6 days prior to their elective procedure if deemed high risk and 4 days if intermediate to low risk (Table 5) (61). Non-ASA NSAID discontinuation prior to spinal procedures is based on the pharmacokinetics of each agent and their respective half-life (Table 6). Commonly used NSAIDs like ibuprofen have a half-life of 5–6 h and is recommended to be discontinued for 1 day prior to spinal intervention (63). These recommendations are similar for Diclofenac and Ketorolac (64, 65). Indomethacin and etodolac have slightly higher half-lives and are recommended to be discontinued for at least 2 days prior to spinal procedure (66, 67). NSAIDS with longer half-lives, meloxicam and naproxen, are recommended to be stopped at least 4 days prior to spinal procedure to ensure a decreased risk of intraprocedural bleeding complication (68, 69). Regarding timing of therapy restoration, the ASRA Pain Medicine recommends restarting these agents within 24 h after their procedure (Table 6) (61).

**Table 6:** Summary of antiplatelet and anticoagulation discontinuation and restarting intervals adapted from the ARSA recommendations and guidelines.

Drug	Discontinuation interval day(s)/hours			Restarting interval
	High-risk	Intermediate-risk	Low risk	
Aspirin	6 days	^a^	None	24 h
Diclofenac	1 days	0 days	0 days	24 h
Ketorolac	1 days	0 days	0 days	24 h
Ibuprofen	1 days	0 days	0 days	24 h
Etodolac	2 days	0 days	0 days	24 h
Indomethacin	2 days	0 days	0 days	24 h
Naproxen	4 days	0 days	0 days	24 h
Meloxicam	4 days	0 days	0 days	24 h
Warfarin	5; INR≤1.2	5; INR≤1.2	0 days	6 h
IV Heparin	6 h	6 h	6 h	2 h
SQ Heparin	24 h	6 h	6 h	2 h—Low risk 6–8 h—Med/high risk
LMWH PPx	12 h	12 h	12 h	4 h—Low risk 12 h—Med/high risk
LMWH Therapeutic	24 h	24 h	24 h	4 h—Low risk 12 h—Med/high risk
Fondaparinux	4 days	4 days	^a^	6 h—Low risk 24 h—Med/high risk
Dabigatran	4 days/6 days RI	4 days/5–6 days RI	^a^	24 h
Rivaroxaban	3 days	3 days	^a^	24 h
Apixaban	3 days	3 days	^a^	24 h
Edoxaban	3 days	3 days	^a^	24 h

Summary of antiplatelet and anticoagulation discontinuation and restarting intervals prior to interventional pain procedure. RI, renal impairment.

^a^ Shared decision making and risk assessment with providers.

### Warfarin

Although warfarin is an older anticoagulant, its use is still prominent among the general population. Warfarin inhibits the *γ*-carboxylation of the vitamin K–dependent coagulation factors (II, VII, IX, and X) and proteins C and S. To verify and monitor its efficacy, INR testing is performed to ensure therapeutic index. According to the ASRA, patients are to stop taking their warfarin 5–6 days prior their planned high-risk or intermediate-risk procedure date with a normalized INR (≤1.2) (70). For low risk procedures, shared decision making with the patient's physicians’ can assess discontinuation protocol, however the ASRA supports continuation in the presence of a therapeutic INR (<3.0) (Table 6) (61).

### Heparin

Heparin is a commonly used anticoagulant that induces its effect by the heparin-mediated inhibition of activated factor Xa. Low-doses, 5000 units every 8–12 h, are commonly given in hospital settings to provide deep vein thromboembolism (DVT) prophylaxis (71). Ideally, patients should undergo discontinuation of subcutaneous heparin for at least 24 h with normalization of aPTT, especially for high risk procedures like kyphoplasty or vertebroplasty. However, for patients on BID or TID dosing, inter-mediate risk procedures can be conducted 6 h after their heparin dose. Post-procedural continuation of heparin can be restarted 2 h after low-risk procedures and 6–8 h after intermediate and high-risk procedures (Table 6) (61).

### LMWH

With a higher and more predicable bioavailability when compared to heparin, low-molecular dose heparin (LMWH) exhibits its antithrombotic effects in a dose-depended manner making laboratory monitoring unnecessary (72, 73). A common commercially available LMWHs is enoxaparin and can be assessed using anti-factor Xa activity level. Its use is prominent for prevention of DVTs in the hospital setting. For low-, intermediate-, and high-risk interventional spine procedures, it is recommended that LMWH is discontinued 12-hours prior spine intervention when using a prophylactic dose. However, when using a 1 mg/kg therapeutic dose, the ASRA recommends a 24-hour interval between discontinuation and the pain intervention, regardless of risk. LMWH can be resumed 4-hours after low-risk procedures and at least 12-hours after intermediate- and high-risk spine procedures (Table 6) (61).

### Fondaparinux

Fondaparinux is another anticoagulant whose mechanism inhibits factor Xa with a half-life of 17–21 h, allowing for daily dosing (74). Due to its long half-life, the ASRA recommends a 5-half-life or 4-day interval of discontinuation prior to intermediate- or high-risk procedures and can be restarted within 24-hours post procedure. Shared management between providers and risk stratification is warranted for low-risk spinal procedures regarding discontinuation, however, a 2-day interval is likely adequate according to the ASRA. Patients undergoing a low-risk spinal procedure may restart their fondaparinux 6 h post procedure (61, 75, 76).

### New oral anticoagulants

New oral anticoagulants (NOACs) such as dabigatran, rivaroxaban, apixaban, and edoxaban are favored over warfarin due to the lack of coagulation monitoring and shorter half-lives. Although this makes NOACs safer, they are more expensive, and their short half-lives mean that missed doses can increase the risk of venous thromboembolism (VTE) (77, 78). Recently, specific antidotes to reverse their effects have been approved or are in clinical trials.

Dabigatran, a direct thrombin inhibitor, binds to thrombin (factor IIa), and prevents the conversion of fibrinogen to fibrin, leading to absence of clot formation (79–81). With a half life- of 14–17 h, dabigatran has been shown to be effective in the prevention of stroke in patients with nonvalvular atrial fibrillation. Moreover, approximately 80% of the drug is renally cleared, thereby increasing its half-life from 14 h to 28 h in patients with end stage renal disease and being contraindicated when patients have a creatinine clearance less than 30 ml/min (82). The current ASRA guidelines reflect this notable aspect of dabigatran's pharmacokinetics and have recommended 4 days between discontinuation and the patient's spinal procedure for intermediate or high-risk pain procedure. For low-risk procedures, 2 days may be considered when shared assessment, risk stratification, and management decision making among providers are followed. For patients with end-stage renal disease, it is recommended that the discontinuation of dabigatran start 5 to 6 days prior to their planned procedure date (61).

Other NOACs, like rivaroxaban, apixaban, and edoxaban are factor Xa inhibitors and has been showed to be as effective as enoxaparin in treatment of venous thromboembolism and non-inferior to warfarin for embolic stroke prophylaxis in patients with atrial fibrillation (79, 83–86). For these three NOACs the ARSA recommends discontinuation 3 days prior to the patients spine procedures to prevent bleeding complications. Similarly to dabigatran, it is safe and recommended to reinitiate 24 h post procedure (61).
